# Structural insights into subunit exchange inhibition and ligand‐induced dimerization of SARS‐CoV‐2 main protease

**DOI:** 10.1002/pro.70874

**Published:** 2026-11-11

**Authors:** Syuan‐Ting Kuo, Zhenyu Xi, Yan J. Sheng, Shiqing Xu, Wenshe Ray Liu, David H. Russell, Xin Yan

**Affiliations:** ^1^ Department of Chemistry Texas A&M University College Station Texas USA

**Keywords:** hydrogen‐deuterium mass spectrometry, main protease, molecular dynamics simulation, native mass spectrometry, subunit exchange

## Abstract

The SARS‐CoV‐2 main protease (M^pro^) is an essential homodimeric cysteine protease that mediates viral polyprotein processing and represents a primary therapeutic target for COVID‐19 treatment. While peptidomimetic inhibitors such as nirmatrelvir have demonstrated clinical efficacy, their effects on M^pro^ quaternary structure dynamics remain poorly understood. Emerging evidence suggests these compounds may paradoxically promote dimerization, a phenomenon termed ligand‐induced dimerization (LID), which could enhance catalytic activity at low inhibitor concentrations. Here, we employed an integrated mass spectrometry platform combining metabolic ^15^N labeling with native mass spectrometry (nMS), hydrogen‐deuterium exchange mass spectrometry (HDX‐MS), and molecular dynamics (MD) simulations to elucidate the dynamic nature of M^pro^ dimerization and its modulation by peptidomimetic inhibitors. We demonstrate that apo‐M^pro^ exists in a dynamic monomer–dimer equilibrium characterized by rapid subunit exchange. Remarkably, peptidomimetic inhibitors, including nirmatrelvir, GC376, and MPI8, kinetically trap the dimeric state, suppressing subunit exchange and stabilizing the dimeric form of M^pro^. Region‐resolved HDX‐MS revealed that inhibitor binding induces widespread conformational stabilization extending beyond the active site to the N‐finger (residues 1–17) and dimer interface (residues 291–302). A notable transition of exchange mode from bimodal (EX1) to unimodal (EX2) in the N‐finger region provided a sensitive structural marker of ligand‐induced dimerization. Molecular dynamics simulations confirmed that the observed stabilization arises primarily from dimer formation rather than direct ligand contacts. These findings establish a mechanistic basis for inhibitor‐stabilized dimerization, identify the N‐finger as a dynamic reporter of quaternary structure transitions, and demonstrate the power of integrated mass spectrometry for dissecting complex protein assembly dynamics with therapeutic implications for antiviral drug development.

## 1 INTRODUCTION

Ligand binding can alter protein conformational ensembles and shift oligomerization equilibria, coupling molecular recognition to changes in quaternary structure and biological activity (Amaral et al., 2017; Ayaz et al., 2023; Daniels et al., 2015). A central consequence of this coupling is ligand‐dependent modulation of subunit exchange, which governs the stability and functional states of many oligomeric protein complexes. This phenomenon is particularly relevant to viral proteases whose catalytic activity depends on dynamic assembly states (Goyal & Goyal, 2020). The severe acute respiratory syndrome coronavirus 2 (SARS‐CoV‐2), the virus responsible for COVID‐19, has caused millions of deaths worldwide and continues to threaten global health and economic stability (Zhu et al., 2020). Its approximately 30,000‐nucleotide RNA encodes 16 non‐structural proteins (nsps) within the ORF1ab polyprotein, which must undergo extensive proteolytic processing to generate the viral replication machinery (Chan et al., 2020; Chen et al., 2020). This processing is mediated by two viral cysteine proteases: the papain‐like protease (PL^pro^), which cleaves three sites, and the main protease (M^pro^, 3CLpro), which performs 11 cleavage events including autocleavage at its own termini (Frolov et al., 2023; Gao et al., 2020; Jin et al., 2022; Zhang et al., 2020). Because of its essential role in viral maturation, high conservation across coronaviruses, and lack of closely related human proteases with similar substrate specificity, M^pro^ has emerged as a major antiviral target (Raj, 2021).

M^pro^ functions as a homodimer in which catalytic activity is tightly coupled to quaternary structure. Each protomer consists of three domains: Domains I and II form the catalytic β‐barrel architecture containing the H41–C145 catalytic dyad, whereas the C‐terminal helical Domain III regulates dimerization (Kneller et al., 2020). Dimer formation is indispensable for activity because the N‐finger of one protomer engages Domain II of the opposing protomer to stabilize the substrate‐binding pocket and properly orient catalytic residues (Kneller et al., 2020). Perturbation of the N‐terminal region increases interfacial dynamics and promotes dimer dissociation (Butalewicz et al., 2024), while mutations in Domain III can allosterically disrupt oligomerization and impair catalysis despite being distal from the active site (Kovalevsky et al., 2024). These observations demonstrate that M^pro^ activity is governed not only by active‐site chemistry but also by dynamic regulation of its assembly state. Consequently, two complementary therapeutic strategies have emerged: disrupting dimerization through allosteric modulation (El‐Baba et al., 2020; Goyal & Goyal, 2020) and directly targeting the catalytic site using peptidomimetic inhibitors (Algar‐Lizana et al., 2022).

Among currently developed antivirals, peptidomimetic inhibitors have shown the greatest efficacy, with many compounds exhibiting low submicromolar antiviral activity (Allerton et al., 2024). Clinically approved inhibitors such as nirmatrelvir (Paxlovid) and ensitrelvir (Xocova) suppress M^pro^ activity through competitive engagement of the catalytic site (Drożdżal et al., 2021; Greasley et al., 2022; Kovalevsky et al., 2024; Yang, Leeuwon, et al., 2022). However, emerging evidence suggests that active‐site binding can paradoxically stabilize or promote the dimeric state of M^pro^, a phenomenon referred to as ligand‐induced dimerization (LID) (Butalewicz et al., 2024; Higashi‐Kuwata et al., 2023; Paciaroni et al., 2023). Quantitative kinetic models integrating ligand binding and oligomerization have shown that GC376, a reversible covalent inhibitor, strongly promotes dimer formation and exhibits cooperative binding behavior toward the dimeric assembly (Arutyunova et al., 2021). Furthermore, Wang et al. reported that inhibitor‐induced dimerization can generate asymmetric dimers in which one protomer remains catalytically active, leading to enhanced activity at low inhibitor concentrations during dose–response measurements (Wang et al., 2025). These observations challenge the conventional view that inhibition and oligomer destabilization are necessarily coupled and instead suggest that ligand binding reshapes the conformational and oligomerization of M^pro^ in a nontrivial manner. Despite increasing recognition of LID, its mechanistic basis remains unresolved, and multiple models involving asymmetric dimers, conformational locking, and transient intermediates have been proposed to explain the phenomenon (Higashi‐Kuwata et al., 2023; Nashed et al., 2022; Wang et al., 2025).

In this work, we investigate how ligand binding modulates subunit exchange and dimer stability in M^pro^ by characterizing its conformational and oligomeric dynamics under native‐like conditions. Using an integrated native mass spectrometry (nMS) and hydrogen–deuterium exchange mass spectrometry (HDX‐MS) approach, we show that peptidomimetic inhibitors kinetically trap M^pro^ dimerization and suppress subunit exchange. Region‐resolved HDX‐MS further identifies dynamically stabilized structural elements at the dimer interface, including a sensitive conformational marker within the N‐finger region associated with ligand‐dependent stabilization. Complementary molecular dynamics simulations support these findings and provide mechanistic insight into how ligand binding propagates stabilization across the dimer interface. Our results provide a mechanistic insight for ligand‐induced dimerization in M^pro^ and reveal structural determinants that regulate subunit exchange in a dynamically evolving viral protease.

## 2 METHODS

### 2.1 Recombinant M^pro^ protein expression and purification

The pET28a‐His‐SUMO‐M^pro^ construct (Yang, Alex Kuo, et al., 2022) was transformed into *E. coli* BL21(DE3) cells. Transformed cells were cultured at 37°C in 6 L 2×YT medium with kanamycin (50 μg/mL) for 3 h and induced with isopropyl‐D‐1‐thiogalactoside (IPTG) at a final concentration of 1 mM when the OD_600_ reached 0.8. After 3 h, cells were harvested by centrifugation at 12,000 × *g*, 4°C for 30 min. Cell pellets were resuspended in 150 mL buffer A (20 mM Tris, 100 mM NaCl, 10 mM imidazole, pH 8.0) and then lysed by sonication on ice. The lysate was clarified by centrifugation at 35,000 × *g*, 4°C for 30 min. The supernatant was loaded onto a nickel‐chelating column with high‐affinity Ni‐charged resin from GenScript and washed with 10 column volumes of buffer A to remove nonspecifically bound proteins, which was followed by elution using buffer B (20 mM Tris, 100 mM NaCl, 250 mM imidazole, pH 8.0). The protein eluates were subjected to buffer exchange with buffer C (20 mM Tris, 10 mM NaCl, pH 8.0) using a HiPrep 26/10 desalting column (GE Healthcare). The His‐SUMO‐M^pro^ proteins were digested with SUMO protease which was purified according to a published protocol (Qiao et al., 2020) overnight at 4°C. The digested protein was applied to a nickel‐chelating column again to remove the His‐tagged SUMO protease, the His‐SUMO tag, and the uncleaved His‐SUMO‐M^pro^. The tag‐free M^pro^ protein was concentrated and loaded on a size exclusion column, HiLoad 16/600 Superdex 75 pg (GE Healthcare), pre‐equilibrated with buffer E (20 mM Tris, 100 mM NaCl, 1 mM EDTA, pH 7.8). The eluted M^pro^ protein in buffer E was concentrated to 20 mg/mL and stored at −80°C for further use.

For N^15^ M^pro^, the same construct was used and was transformed into *E. coli* BL21(DE3) cells. The transformed cells were cultured in 5 mL LB medium with kanamycin (50 μg/mL) at 37°C overnight. The cells were then collected by centrifugation at 4000 × *g*, 4°C for 5 min. The supernatant was discarded, and the cells were resuspended into 50 mL M9 medium, prepared with ^15^NH_4_ Cl with kanamycin (50 μg/mL), and cultured at 37°C overnight. Following this, 200 mL of M9 medium, prepared with ^15^NH_4_ Cl with kanamycin (50 μg/mL), was added to the cells. The cells were induced with IPTG at a final concentration of 1 mM when the OD_600_ reached 0.6 and were cultured overnight at 16°C. The purification of N15 M^pro^ followed the same process as that of M^pro^.

### 2.2 Native mass spectrometry

The total protein concentration (determined by *A*_280_) was 10 μM unless otherwise specified to ensure dimeric (active) M^pro^ was the major species in the general cases (i.e., less than 5% monomeric M^pro^ was presented in spectra). M^pro^ was buffer exchanged to 200 mM ammonium acetate (pH 6.8) with 6 kD cut‐off spin column (BIO‐RAD) prior to mass spectrometry analysis. Unlabeled and metabolically labeled proteins were each incubated with inhibitors for 30 min at room temperature to allow complex formation. The two samples were then mixed to initiate subunit exchange, which was monitored by time‐resolved mass spectrometry (TRMS). Native mass spectrometry was carried out on a Q‐Exactive Plus UHMR (Thermo Fisher Scientific) with Nanospray Flex ion source (Thermo Fisher Scientific). Spray voltage was set to 1.0–1.5 kV and the temperature of the ion transfer tube was set to 50°C. To remove the non‐specific adducts without disrupting the dimerization, activation energies were carefully optimized as follows: in‐source trapping (IST): −10 V, in‐source dissociation (CID): 10 eV, and higher energy in collision cell (HCD): 20 V. Trapping gas was set to 5 and resolution was 6250 unless otherwise specified.

### 2.3 Hydrogen deuterium exchange mass spectrometry

M^pro^ was buffer exchanged from the storage buffer to working buffer with 20 mM MOPs and 180 mM NaCl (pH 6.8, adjusted by sodium hydroxide).

The labeling buffer was prepared with the same formulation, except the pH was adjusted using sodium hydroxide (NaOH) dissolved in deuterium water (D_2_O). In the labeling step, 2 μL of M^pro^ in H_2_O buffer was labeled by adding 18 μL D_2_O buffer, resulting in H_2_O:D_2_O = 1:9 (v/v, pH = 7.2, 90% D_2_O). The final concentration of M^pro^ was 9 μM for apo and inhibitor‐bound studies unless otherwise specified. The final concentration of Nirmatrelvir was 10 μM. The temperature was controlled at 25°C during the labeling step before being quenched. In the quenching step, labeled M^pro^ was mixed with 8 M guanidine chloride in a 1:1 volume ratio on ice and the final pH of the pre‐calibrated quenched solution was 2.45 to minimize the back‐exchange. Fully deuterated control samples (All‐D) were prepared by incubating M^pro^ in the presence of 7.2 M deuterated urea (urea‐d_4_) with 90% D_2_O (pH_read_ 6.8) at 25°C for 24 h. Deuterium uptakes (max‐D) from the All‐D control samples were used to normalize the uptakes in each region to account for back‐exchange (Lantz et al., 2025; Masson et al., 2019). The sample was digested by a home‐packed pepsin column followed by a five‐minute trapping step on the C8 trap column (5 μm 5 × 1.0 mm, Higgins Analytical). Peptides were separated by a C18 analytical column (3 μm 50 × 1.0 mm, Higgins Analytical) on the Agilent 1290 HPLC system. The eluent compositions were A: 0.1% formic acid (FA), 0.05% trifluoroacetic acid (TFA) in water (pH 2.3), and B: 0.1% FA, 0.05% TFA in acetonitrile (ACN). The elution gradient was set from 10% B to 65% B in 25 min followed by a 5‐min cleaning step with 90% B. The injection and separation were done in a modified chill box equipped with two six‐way injection valves with controlled temperature (0–0.5°C). The mass spectrometry analysis was done on the Agilent 6545XT. The peptide mapping was done with BioConfirm 10.0 (Agilent). The MS1 and MS2 mass accuracy for peptide identification was set to 10 and 20 ppm, respectively. Deuterium uptakes on individual peptides at different time points were extracted by ExMS2 (Kan et al., 2019).

### 2.4 Simulation method and system setup

Replica exchange with solute tempering (REST) was applied to sample the N‐finger conformers (Liu et al., 2005; Wang et al., 2011). The initial structures of the apo monomer and dimer were both derived from PDB entry 7JPY (Yang et al., 2021), while the holo dimer structure was obtained from PDB entry 7TE0 (Yang et al., 2021). The monomer was placed in an orthorhombic simulation box, ensuring a minimum buffer distance of 10 Å from the protein to the box edges to prevent boundary effects. The system was solvated with 11,325 TIP3P (Mark & Nilsson, 2001) water molecules and neutralized with 4 Na^+^ counterions. Periodic boundary conditions were applied to mimic an infinite system environment. Residues 1–17 were defined as the “solute” region subjected to Hamiltonian scaling and tempering. The remaining regions of the system were maintained at a single temperature, ensuring that only the solute region experienced temperature fluctuations. The simulation was conducted using the OPLSAA force field (Kaminski et al., 2001) as an NPT ensemble at a pressure of 1.01325 bar for 100 ns, with all setup performed in Desmond (D.E. Shaw Research, New York, NY, 2024) (Bowers et al., 2006). REST was implemented with eight replicas at temperatures of 300, 323.3, 347.7, 374.6, 402.6, 432.8, 464.5, and 498.3 K. This temperature ladder was optimized to achieve an exchange acceptance ratio of 29.8%, balancing efficient replica exchanges with computational efficiency. Solvent‐accessible surface area (SASA) was calculated using MDAKit (Mitternacht, 2016). To characterize the dominant conformational motions of the N‐finger region, principal component analysis (PCA) was performed on the Cα coordinates of residues 1–17; trajectory frames were superimposed onto the average structure to remove overall translation and rotation, and the aligned coordinates were mean‐centered across all 1000 frames prior to PCA. Details of dimensionality reduction are reported in Appendix S1.

## 3 RESULTS

### 3.1 Subunit exchange of dimeric M^pro^ characterized by metabolic labeling‐nMS

As the intactness of M^pro^ termini is important for dimerization, traditional biophysical approaches requiring modifications on N‐ or C‐termini (i.e., SPR, chemical labeling, and genetically encoded tags) could hinder reliable biophysical readouts (Johansen‐Leete et al., 2022; Zhong et al., 2008). To address this limitation, we incorporated metabolic labeling with high‐resolution native mass spectrometry (nMS) to investigate the dimerization process.

Metabolic labeling was achieved by supplying ^15^N as the sole nitrogen source during cell culture, resulting in full incorporation of ^15^N into the expressed protein in place of the naturally abundant ^14^N. This approach preserves the structural integrity of M^pro^ while introducing a defined mass shift that distinguishes unlabeled (light, L) from labeled (heavy, H) subunits. The mass‐resolved isotopic tracer allows two scenarios to be distinguished. When the dissociation rate of M^pro^ is negligible, only two homodimeric species (i.e., L–L and H–H) are expected to be observed in the mass spectra. Conversely, when the dissociation rate of M^pro^ occurs on an experimentally accessible timescale, monomeric subunits (i.e., 2 L from L–L or 2H from H–H) are released and subsequently reassociate to form heterodimers (i.e., H–L); the detection of these heterodimeric species thus indicates the dynamic interchange of monomeric and dimeric M^pro^ in solution (Figure 1a).

**FIGURE 1:**
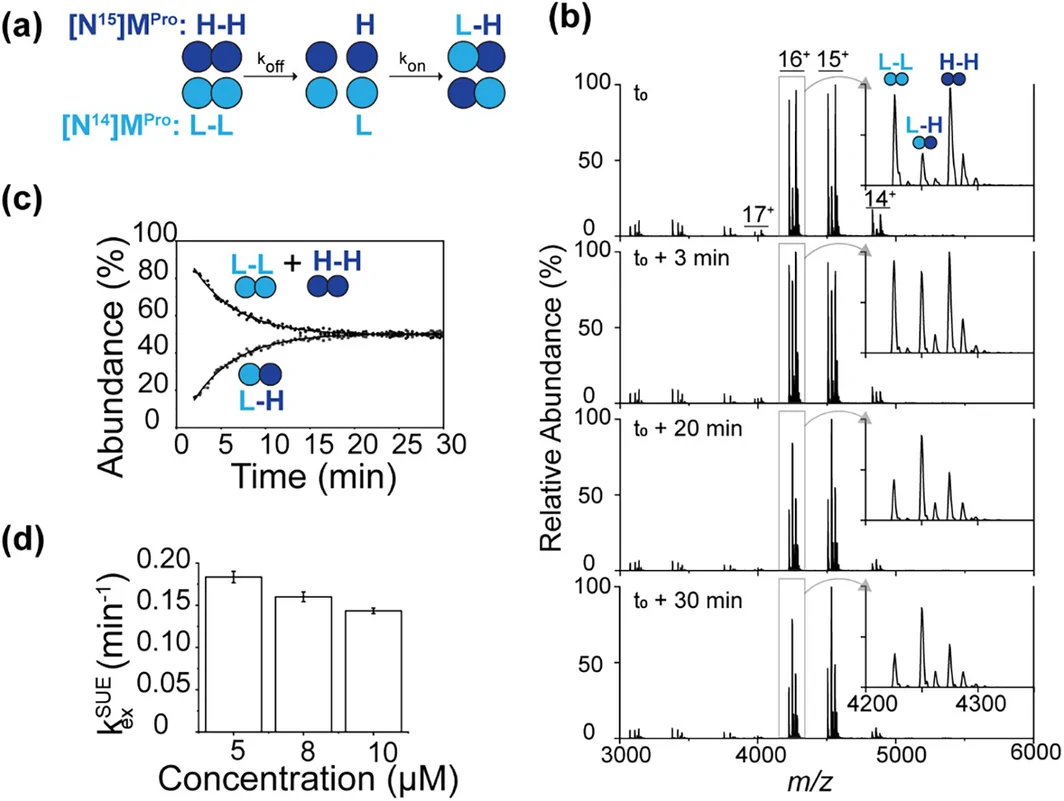
Subunit exchange (SUE) between unlabeled and ^15^N isotopically labeled M^pro^ (a) Schematic representation of the subunit exchange process. (b) Representative time‐resolved native mass spectra of unlabeled M^pro^ (U) mixed with ^15^N isotopically labeled M^pro^ (L) at a 1:1 ratio. The initial time point (*t*_0_, ~3 min) reflects the sample loading time into the capillary and was considered in the calculation of kinetic constants. (c) Temporal changes in the relative abundance of homodimers (U–U + L–L) and heterodimers (U–L). (d) Effect of total protein concentration on the subunit exchange rate constant of dimeric M^pro^.

Immediately after mixing, the mass spectra were dominated by homodimers (L–L, 67,600 Da; H–H, 68,390 Da), consistent with the full substitution of ^14^N with ^15^N across 402 nitrogen atoms per monomer (Figure 1b, *t*_0_). A post‐translational modification (PTM) with a mass shift of 183 Da was observed in the isotopically labeled M^pro^. This modification is attributed to the expression conditions in *E. coli*, as the reduced protein expression rate inherent to M9 medium during metabolic labeling may render the protein more susceptible to frequently occurring PTMs (Brown et al., 2017). For all subsequent analyses, the contribution of this species was accounted for in kinetic determination (*k* ^sub^_ex_).

In our static spray experiment, the dead time (*t*_0_, ca. 3 min) represented the interval from sample loading to the onset of a stable spray signal, which was incorporated into the calculation of *k*_ex_. Within 6 min of mixing (Figure 1b), *t*_0_ + 3 min, the heterodimer population increased from ~20% to ~80%, accompanied by a corresponding decrease in homodimers. The gradual progression confirmed that subunit exchange could be resolved in real time, rather than collapsing instantaneously to equilibrium. After ~20 min, the system approached the statistical distribution of 1:2:1 for homodimers and heterodimers (Figure 1b, *t*_0_ + 20 min, *t*_0_ + 30 min). Kinetic fitting yielded an exchange rate constant of 0.18 ± 0.01 min^−1^, corresponding to a dimer half‐life of 3.85 ± 0.02 min (Figure 1c) (Thangaraj et al., 2019).

To probe the contribution of monomeric species, the protein concentration was reduced from 10 to 5 μM, thereby increasing the proportion of monomers in solution. Under these conditions, the subunit exchange rate accelerated by ~1.2‐fold with statistical significance (*n* = 3, *p* ≤ 0.05, Figure 1d). The observation that a two‐fold reduction in concentration increased the exchange rate indicates that the observed kinetics are dominated not by dimer concentration; otherwise *k* ^sub^_ex_ would instead slow down upon dilution. Alternatively, the exchange kinetics increased due to a higher monomer fraction, supporting a reversible dissociation‐reassociation mechanism where the dissociation step (*k*_off_) is the rate‐limiting step, followed by a fast reassociation rate (*k*_on_) proportional to monomer ratio (Chen et al., 2014). The observed subunit exchange rate, a combined result from *k*_off_ and *k*_on_, therefore principally represents *k*_off_, whereas independent determination of *k*_on_ requires further kinetic studies or the measurement of the equilibrium constant (*K*_d_).

### 3.2 Peptidomimetic inhibitors suppress subunit exchange and stabilize the dimer

The verification of subunit exchange (SUE) in M^pro^ dimerization established that the enzyme exists in a dynamic equilibrium rather than a static distribution of monomers and dimers. This finding prompted us to investigate how peptidomimetic inhibitors perturb this equilibrium. We therefore examined the effects of MPI8, GC376, and nirmatrelvir by incubating 10 μM M^pro^ (L or H) with 20 μM inhibitor (v/v = 50/50, resulting in a final concentration of 5 μM protein and 10 μM inhibitors). In all cases, mass spectra confirmed the formation of the [M + 2I] complex (Figure S1).

In contrast to the apo enzyme, which reached equilibrium within minutes (Figure 2a), subunit exchange was nearly suppressed upon inhibitor binding. MPI8‐complexed M^pro^ exhibited no detectable heterodimers even after 12 h of incubation (Figure 2b). To test the universality of observation, we further included GC376 and nirmatrelvir under prolonged incubation (20 h) and observed similarly strong inhibition of SUE, with only trace heterodimer formation (Figure 2c,d). The trace heterodimer signal was quantified at less than 2% of the total dimer‐containing species with all three peptidomimetics investigated, a level significantly lower than the contribution from the early timepoint of inhibitor‐unoccupied proteins (Figure 2a, ~20% within 6 min).

**FIGURE 2:**
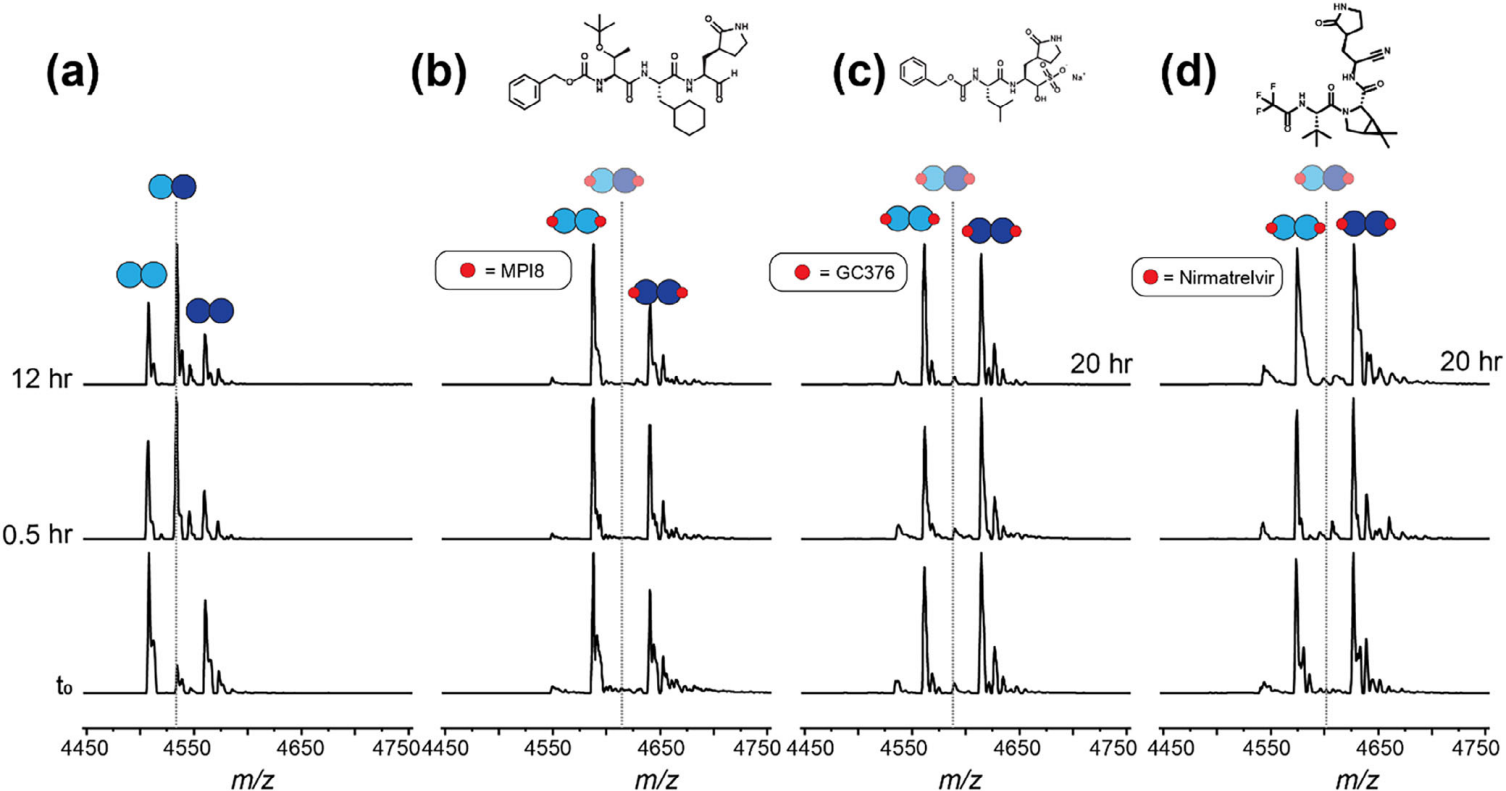
Time‐evolved subunit exchange profiles for (a) apo‐M^pro^ and M^pro^ bound to (b) MPI8, (c) GC376, and (d) Nirmatrelvir. Dashed gray lines indicate the expected *m*/*z* of the heterodimer. Observed mass shifts agree with the expected mass shifts caused by inhibitor binding (see Table S1 for expected and experimental masses). Light blue and dark blue solid circles denote unlabelled and ^15^N‐labeled M^pro^, respectively.

This suppression of exchange, rather than any loss of inhibitor affinity, accounts for the species observed in our spectra. The absence of inhibitor‐bound heterodimers does not indicate that inhibitors fail to engage the heterodimeric species; because the two active sites are structurally equivalent irrespective of isotopic labeling, pre‐existing heterodimers would be expected to bind inhibitor normally. Instead, L/H‐inhibitor complexes are not observed because heterodimers are largely not generated once inhibitor‐bound homodimers become kinetically trapped, such that exchange rather than binding is the limiting event.

### 3.3 Nirmatrelvir induces widespread and localized conformational stabilization detected by HDX‐MS

As metabolic labeling with nMS demonstrated that ligand‐induced dimerization (LID) arises from inhibition of subunit exchange, we next sought to determine how this stabilized dimeric state alters the regional dynamics of M^pro^ with residue‐level resolution. HDX‐MS under two conditions was performed to maintain consistency with our native MS experiments: (i) 9 μM apo‐M^pro^ and (ii) 9 μM M^pro^ in the presence of 10 μM nirmatrelvir, with labeling times ranging from 10 s to 3 days to fully capture the experimental dynamic range (Masson et al., 2019; Ozohanics & Ambrus, 2020).

Full sequence coverage enabled evaluation of exchange profiles across the entire protein (Figure S2 and Table S1). We observed both consistent (e.g., peptide 219–229) and reduced (e.g., peptide 35–44) deuterium uptake profiles across labeling times ranging from 10 s to 3 days, capturing the complete kinetic profiles of nirmatrelvir‐bound M^pro^ (Figure 3a). Notably, we do not assign statistical significance to individual peptides. Our interpretation is instead qualitatively confined to differences exceeding 20% in deuterium uptake at three or more labeling time points. The peptides spanning residues 209–287, for example, remain below this threshold at every time point and were therefore not interpreted as reporting altered structural fluctuation. The results were represented in a heatmap with regions of reduced uptake upon inhibitor binding, shown in blue, corresponding to locally restricted dynamics relative to the apo state (Figure 3b).

**FIGURE 3:**
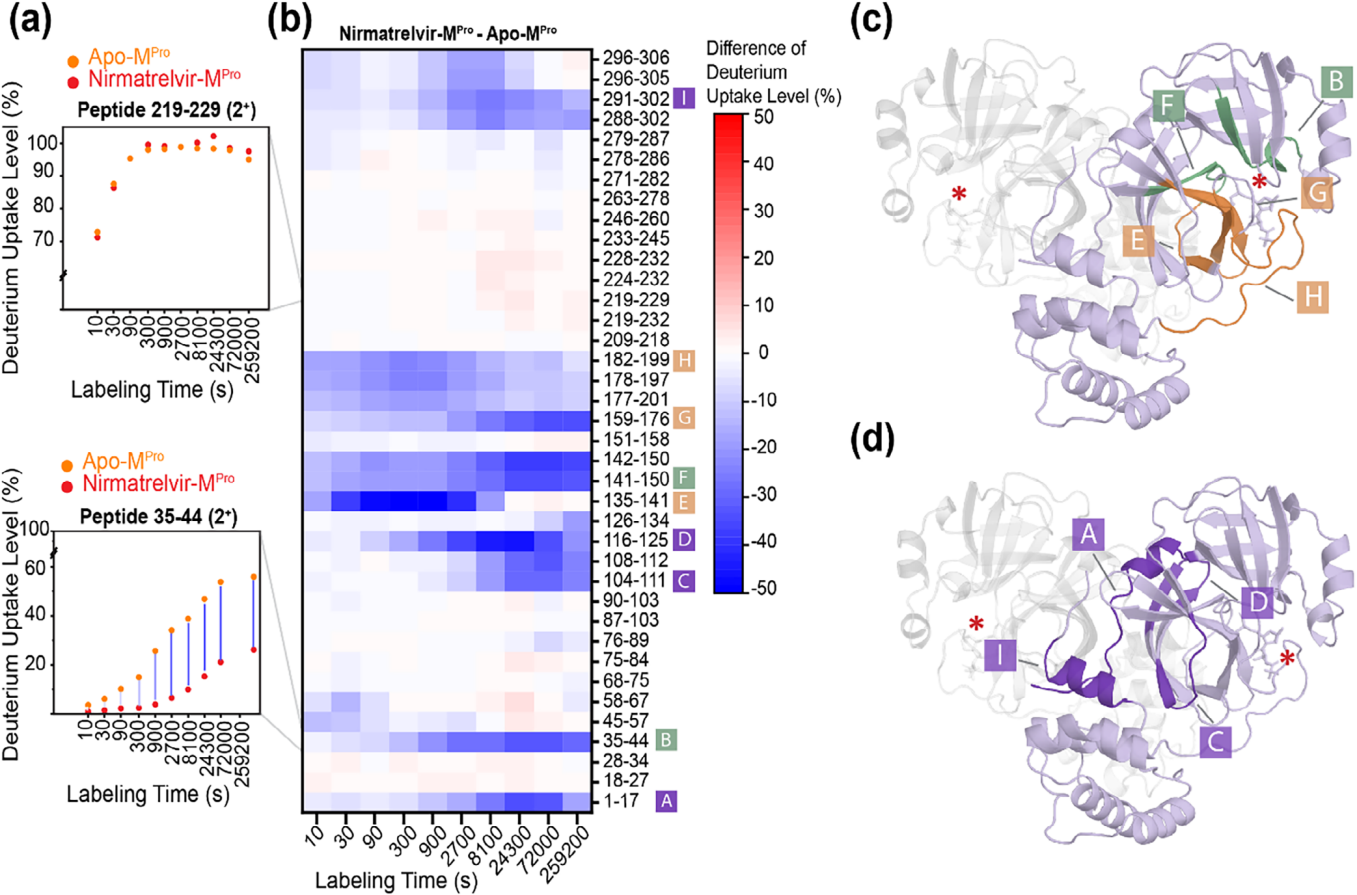
(a) Representative deuterium uptake profiles for peptides 219–229 and 35–44. Deuterium uptake is normalized to the fully deuterated state. (b) Heatmap comparing deuterium exchange in the M^pro^‐Nirmatrelvir complex versus apo‐M^pro^. Negative values (blue) indicate reduced deuterium exchange upon binding of Nirmatrelvir. (c) Residues in direct contact with nirmatrelvir and (d) residues without direct contact are mapped onto the crystal structure (PDB: 7TE0).

From the heatmap, we noted distinct temporal patterns of protection across labeling times. Peptides spanning around the covalent modification site (Cys145) show a strong reduction in uptake (blue) that appears already at the earliest labeling times and persists throughout the time course, consistent with an immediate perturbation of the amide hydrogen‐bonding network upon covalent bond formation. On the other hand, for instance, peptide 35–44, which contains the catalytic His41 and lies in direct non‐covalent contact with nirmatrelvir, instead shows minimal uptake differences at early timepoints (10–90 s) that only intensify into a comparable reduced uptake at longer labeling times (i.e., >900 s), a lagged separation between apo and complex uptake profiles. While this interpretation is correlative and requires further investigation, we interpret this lagged reduced uptake reflecting a slow and gradual rigidification of the local hydrogen‐bonding network compared to the immediate constraint imposed by proximity of the active site.

The heatmap revealed a broad reduction in deuterium uptake spanning peptides 104–111 through 182–199, overlapping with Domain II, which forms the catalytic core responsible for substrate proteolysis. Mapping these results onto the reported crystal structure allowed us to distinguish altered dynamics arising from direct contact with inhibitors or potential allosteric modulations. To map on structures, we adopted contact thresholds used in structural studies of protease‐inhibitor complexes by defining direct contact as any residue with at least one non‐hydrogen atom within 4.0 Å of nirmatrelvir in the crystal structure (PDB: 7TE0). Peptides including 35–44, 142–150, 159–176, and 182–199 exhibited reduced uptakes which are likely due to their direct contact with nirmatrelvir (Figure 3c). Notable regions including peptides 1–17, 104–111, 116–125, and 291–302 showed reduced uptake despite lacking direct interactions with nirmatrelvir in the crystal structure (Figure 3d). Importantly, peptide 291–302 is located at the dimer interface, where Arg298 is known to regulate M^pro^ dimerization. This particular region therefore offered strong and supporting evidence of ligand‐induced dimerization with region‐specific resolution.

### 3.4 The N‐finger as a dynamic reporter of ligand‐induced dimerization

While EX2 exchange kinetics are more common than EX1 kinetics, we observed an EX1 exchange mode in the N‐finger region of apo‐M^pro^. The bimodal distribution progressed at the onset time point of 5 min and showed a signature bimodal isotopic distribution (~6 Da separation between two populations) after 45 min of labeling (Figure 4a). The bimodality indicated potential unfolding or significant conformational changes to allow cooperative deuterium uptakes (Linderstrøm–Lang model, Figure S3) (James et al., 2022). The half‐life of this conversion was approximately 110 min (Figure S4), considerably longer than the ca. 4‐min half‐life observed for subunit exchange, thereby ruling out the possibility that the bimodal distribution is a redundant reflection of subunit exchange. Given the coexistence of monomeric and dimeric M^pro^ under this condition, this observed EX1 exchange mode could arise from the monomeric M^pro^, the dimeric M^pro^, or a combination of both.

**FIGURE 4:**
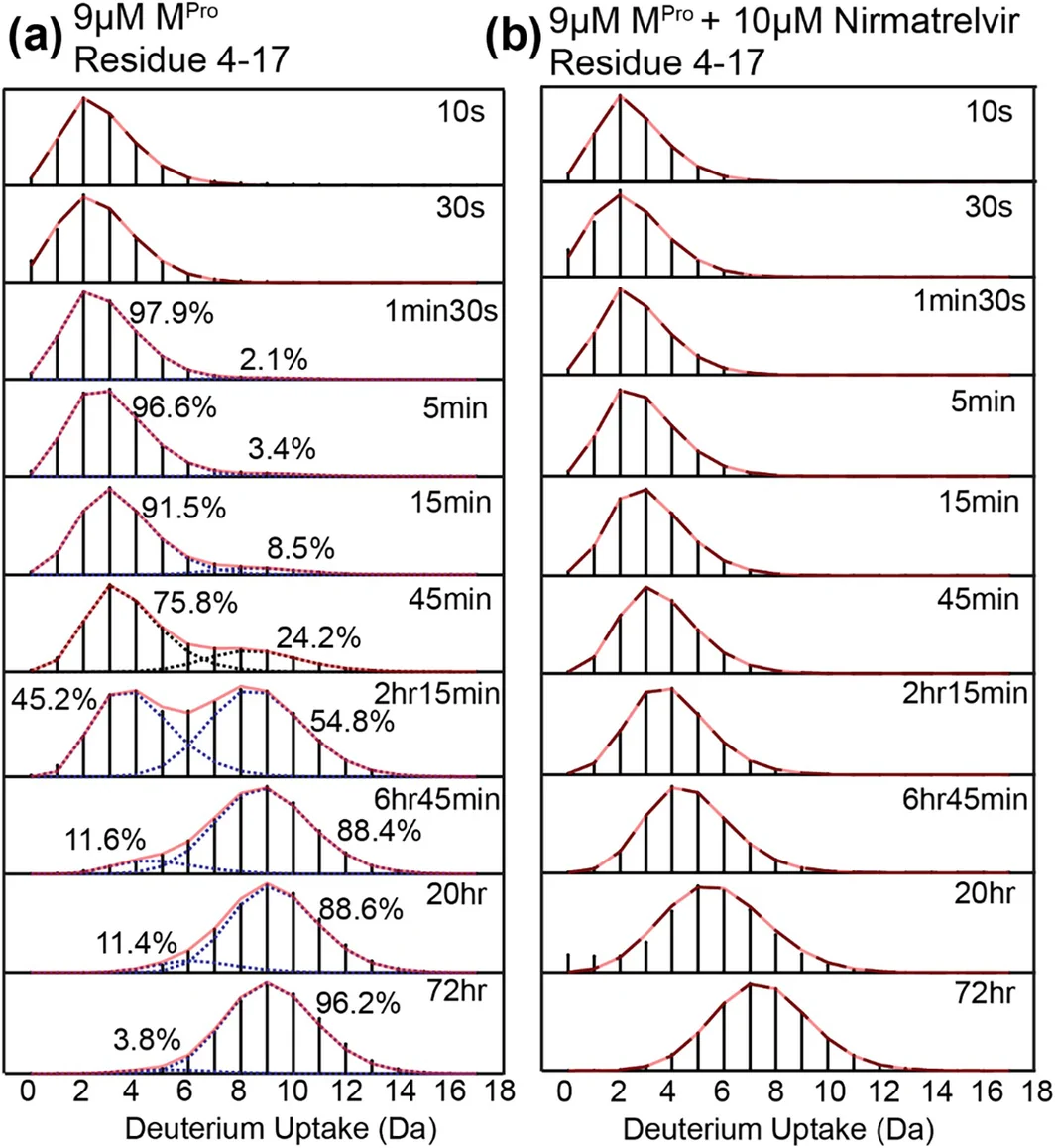
EX1 kinetics identified in peptide region 4–17. (a) Time‐resolved deuterium uptake mass spectra of apo‐M^pro^. The relative abundance of two distinct populations, denoted as low‐deuterium (low‐D) and high‐deuterium (high‐D) uptake populations, was quantified based on their respective areas under the curve. (b) Time‐resolved deuterium uptake mass spectra of M^pro^ in complex with Nirmatrelvir.

Notably, a unimodal distribution was observed in the same region instead of the bimodal distribution when both active sites of dimeric M^pro^ were occupied by nirmatrelvir (Figure 4b). Given that our SILAC‐nMS revealed the inhibition of subunit exchange upon nirmatrelvir binding, this transition from EX1 to EX2 kinetics suggested that the conformational dynamics of N‐finger (exhibiting EX1 kinetics) are most likely attributable to monomeric M^pro^. Consistent with this interpretation, MD simulations showed that residues 1–17 of monomeric M^pro^ exhibited a bimodal distribution along the first principal component (PC1), driven primarily by the positions and reorientations of Arg4, Lys5, and Met6, a feature absent in the dimeric form (Figure S5). To our knowledge, while several HDX‐MS studies have examined M^pro^, the EX1 kinetics of the N‐finger region in apo‐M^pro^ have not been previously reported, as prior studies were conducted with a strong focus on dimeric M^pro^ (Abe et al., 2022; Pagotto et al., 2025).

### 3.5 Molecular dynamic simulations to dissect conformer contributions

HDX‐MS analysis highlighted critical regions of altered dynamics following peptidomimetic binding; however, the relative contributions of ligand‐mediated hydrogen bonding versus dimerization of M^pro^ remain unclear. To deconvolute this information, we performed molecular dynamics (MD) simulations under three conditions: apo monomeric M^pro^ (Figure 5a, orange), apo dimeric M^pro^ (Figure 5a, green), and dimeric M^pro^ with both active sites occupied by nirmatrelvir (Figure 5a, blue). While the simulations were not intended to recapitulate the HDX kinetics, they allowed us to define and observe physiologically inaccessible states (i.e., monomer‐only and dimer‐only states) to identify local differences between them. Structural similarities among these states were evaluated by comparing the solvent‐accessible surface area (SASA) at each residue (Figure 5b,c). The utility of MD lies in its ability to populate a variety of conformational states and delineate their individual characteristics, which is experimentally inaccessible due to the coexistence of monomeric and dimeric M^pro^ under physiological conditions.

**FIGURE 5:**
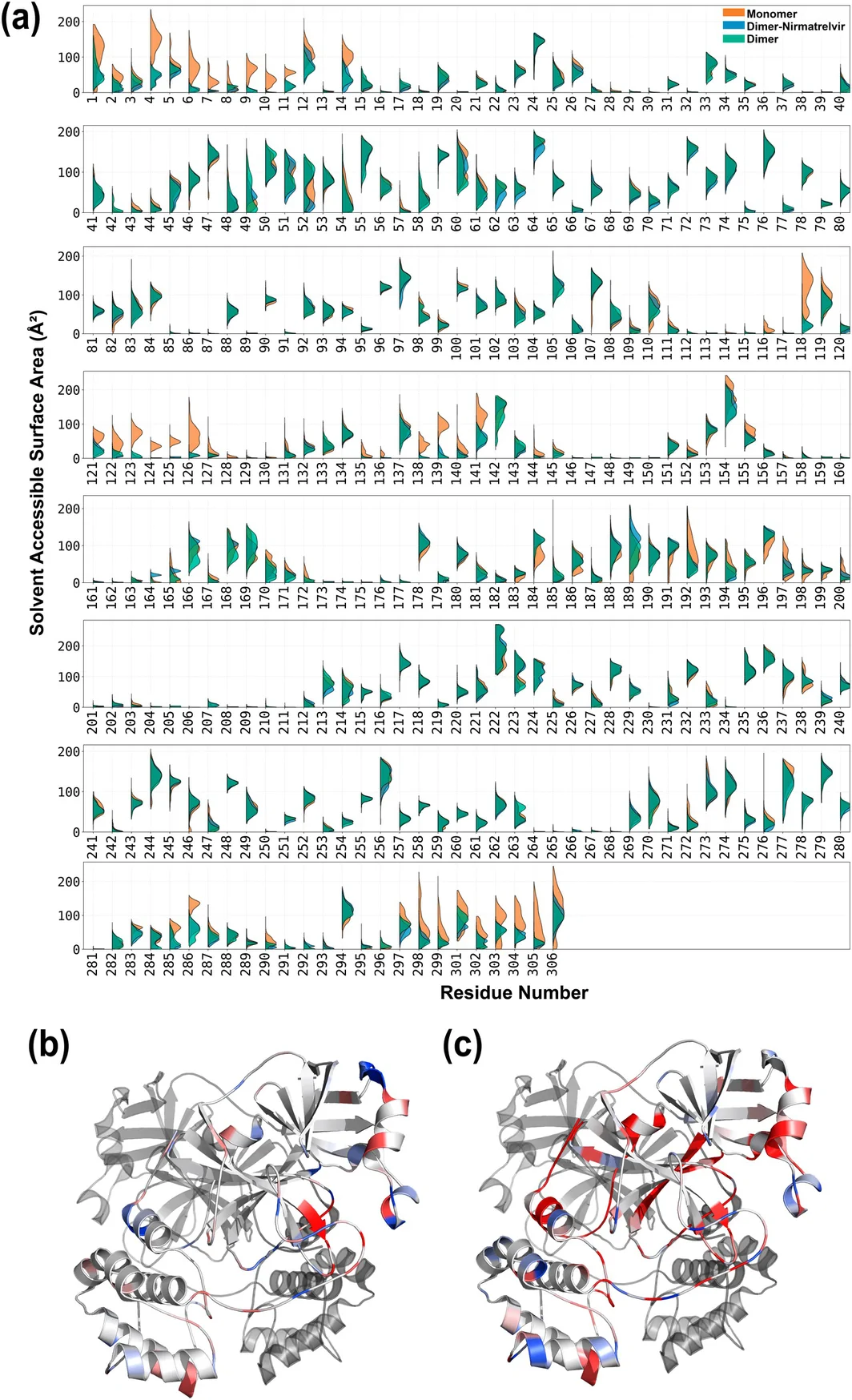
(a) Solvent‐accessible surface area (SASA) distributions for each residue of M^pro^, represented as multiviolin plots, with color coding for the monomeric apo‐M^pro^ (orange), the M^pro^–nirmatrelvir complex (blue), and the apo‐dimeric M^pro^ (green). Distributions were derived from 1000 frames sampled across a 100‐ns simulation trajectory. (b) Per‐residue SASA difference between the M^pro^‐nirmatrelvir dimer and apo‐M^pro^ dimer, mapped onto the crystal structure (PDB: 7TE0). (c) Per‐residue SASA difference between the apo‐M^pro^ monomer and apo‐M^pro^ dimer, mapped onto the crystal structure (PDB: 7TE0). In (b) and (c), red indicates increased SASA and blue indicates decreased SASA relative to the apo‐M^pro^ dimer.

Comparison of SASA profiles revealed two distinct patterns. Across most residues over the 100 ns trajectory, differences in SASA among the monomeric, dimeric, and nirmatrelvir‐bound dimeric states were negligible. In contrast, significant variations emerged within the N‐ and C‐terminal regions, as well as residues 116–155 and 183–200. Significant reductions in SASA were observed in regions both directly contacting nirmatrelvir (e.g., residues 116–155 and 183–200) and distal regions lacking direct ligand interactions (e.g., residues 1–17 and 291–302). The latter are particularly consistent with the reduced HDX observed in regions unlikely to be stabilized through direct ligand contacts, suggesting that dimerization contributes substantially to the observed conformational stabilization. While the monomeric (Figure 5a, orange) profile is distinct, the apo‐dimeric and nirmatrelvir‐bound (Figure 5a, green & blue) profiles show high similarity. These results suggest that the stabilization and reduced dynamics observed via HDX‐MS are primarily driven by M^pro^ dimerization rather than direct hydrogen‐bonding networks established by nirmatrelvir. Therefore, the observed stability and allosteric effects are more closely associated with the quaternary structure than with ligand‐induced interactions.

## 4 DISCUSSION

Ligand‐dependent modulation of oligomerization is a widespread mechanism for regulating protein activity, enabling conformational transitions that couple molecular recognition to biological function (Amaral et al., 2017; Ayaz et al., 2023; Daniels et al., 2015). In receptor tyrosine kinases such as EGFR, ligand binding induces structural rearrangements that expose dimerization interfaces and initiate downstream signaling cascades (Dawson et al., 2005). A similar dependence on quaternary structure is observed in the SARS‐CoV‐2 main protease (M^pro^), where catalytic activity requires formation of a functional homodimer (Noske et al., 2023; Yadav et al., 2022). Although substrate‐ and ligand‐regulated oligomerization is broadly recognized as an important regulatory mechanism, the transient and heterogeneous nature of these assemblies complicates mechanistic characterization. In particular, dynamic exchange between monomeric and dimeric states presents a challenge for ensemble‐based structural and biochemical approaches, especially when ligand binding simultaneously perturbs conformational and oligomeric equilibria (Ferreira & Rabeh, 2020; Fraser & Murcko, 2024; Jochmans et al., 2023; Li et al., 2010; Sonn‐Segev et al., 2020).

Our results support a model in which peptidomimetic inhibitors reshape the conformational ensemble of M^pro^ by stabilizing a kinetically trapped dimeric state that is resistant to subunit exchange. This mechanism provides a structural explanation for the long‐observed phenomenon of LID (Jochmans et al., 2023; Li et al., 2010; Nashed et al., 2022). Rather than functioning only through competitive occupancy of the catalytic site, peptidomimetic inhibitors appear to reinforce interprotomer interactions that suppress dynamic dissociation pathways. This interpretation is consistent with previous kinetic studies reporting biphasic concentration‐response behavior, in which low concentrations of inhibitor transiently enhance catalytic turnover prior to inhibition at higher concentrations (Higashi‐Kuwata et al., 2023). Such behavior has been attributed to asymmetric active‐site occupancy within the dimer, where stabilization of the dimeric state can transiently increase the population of catalytically competent assemblies. Our findings extend this model by suggesting that once both active sites are occupied, the dimer becomes conformationally stabilized and subunit exchange is effectively suppressed, thereby preventing formation of dynamically exchanging asymmetric dimers.

Two aspects of this model warrant further investigation. In the present study, inhibitors were pre‐incubated with each M^pro^ population before mixing, a design chosen to test whether inhibitor‐bound dimers remain exchange competent; in one future direction, inhibitor addition to a pre‐mixed sample at different time points would reveal whether inhibitor binding arrests an actively exchanging population immediately or attenuates exchange progressively. In addition, our experiments employed inhibitor concentrations sufficient for near‐complete occupancy of both active sites, conditions under which singly occupied asymmetric dimers are not appreciably populated. Similar TRMS with sub‐stoichiometric inhibitor concentrations will allow detection of asymmetric dimers to rationalize whether a single bound inhibitor is sufficient to confer kinetic trapping, or whether occupancy of both sites is required for stabilization to propagate across the dimer interface.

The comparison between peptidomimetic inhibitors and halicin further highlights the importance of interprotomer stabilization in this process. We selected a panel of three peptidomimetic inhibitors, MPI8, GC376, and nirmatrelvir, that differ substantially in their electrophilic warhead chemistry: MPI8 contains an aldehyde that forms a reversible hemithioacetal, GC376 is a dipeptidyl aldehyde bisulfite adduct prodrug that converts into its active aldehyde form, and nirmatrelvir contains a nitrile that forms a reversible thioimidate, all of which target the catalytic cysteine (Cys145). Despite differences in their covalent‐bond‐forming chemistries, all three inhibitors share an extended substrate‐mimicking binding mode, engaging multiple subsites (S1–S4) of the M^pro^ active site in a manner that resembles natural substrate engagement. This chemical diversity allowed us to decouple the specific mode of covalent reactivity from the shared feature of substrate‐mimetic occupancy. If suppression of subunit exchange were a direct consequence of substrate‐mimetic binding within the active site, rather than a distinct property of any single inhibitor's chemistry, we reasoned that this effect should be observed consistently across all three inhibitors despite differences in their warheads. Indeed, all three inhibitors suppressed subunit exchange to a similar extent, supporting a generalizable, binding‐mode‐dependent mechanism rather than an inhibitor‐specific effect. Halicin (Yang, Alex Kuo, et al., 2022), a nitrothiazole‐containing covalent inhibitor, did not suppress subunit exchange (Figure S6). This provides plausible evidence that ligand‐induced dimer stabilization arises not simply from catalytic‐site engagement, but from redistribution of conformational equilibria toward a structurally reinforced dimeric state. These results provide a basis for ligand‐induced dimerization (LID): occupation of the active sites by peptidomimetic inhibitors kinetically traps M^pro^ in the dimeric form, thereby preventing dissociation into monomers and shifting the equilibrium toward the dimer population.

Our HDX‐MS and molecular dynamics analyses further identify structural features associated with this stabilization mechanism, with each technique probing a distinct but complementary layer of structural change: HDX‐MS reports on backbone amide solvent protection, while MD‐derived SASA metrics capture side‐chain accessibility. Previous mutational studies demonstrated that substitutions including S1A, S10T, S139A, E166A, and E166V disrupt dimerization, reduce catalytic activity, or confer resistance to nirmatrelvir through perturbation of interprotomer hydrogen‐bonding networks (Duan et al., 2023; Ferreira et al., 2022; Iketani et al., 2022). Consistent with these findings, our data indicate that dimerization precedes high‐affinity peptidomimetic binding. The apo‐dimer and inhibitor‐bound dimer exhibited highly similar SASA profiles relative to the pronounced differences observed between monomeric and dimeric states, suggesting that the active‐site geometry required for inhibitor binding is preorganized through dimer formation rather than induced by ligand binding itself. Accordingly, the monomer‐bound intermediate likely contributes minimally to the inhibition pathway. Reduced susceptibility of monomer‐rich mutants toward nirmatrelvir can therefore be rationalized by insufficient formation of the preorganized dimeric assembly required for stable inhibitor engagement.

A particularly notable finding is the ligand‐dependent stabilization of the N‐finger region and the transition from bimodal (EX1‐like) to stabilized exchange behavior upon inhibitor binding (Figure S7). These observations suggest that the N‐finger serves as a sensitive structural reporter of interprotomer stabilization and may represent a key determinant governing the dynamic equilibrium between exchange‐competent and kinetically trapped dimers. More broadly, our findings emphasize that active‐site ligands can regulate enzymatic activity not only through direct catalytic inhibition, but also through modulation of quaternary structure and subunit exchange dynamics. From a therapeutic‐design perspective, inhibitor optimization may therefore consider not only active‐site affinity but also the ability to stabilize a non‐exchanging, catalytically inhibited dimeric state.

## 5 CONCLUSION

We establish a mechanistic basis for ligand‐induced dimerization in SARS‐CoV‐2 M^pro^ by demonstrating that peptidomimetic inhibitors stabilize a kinetically trapped dimeric state and suppress subunit exchange. Integration of native mass spectrometry, HDX‐MS, and molecular dynamics simulations revealed structural regions associated with ligand‐dependent stabilization, particularly within the dimer interface and N‐finger domain. These findings indicate that inhibition of M^pro^ involves not only catalytic‐site occupancy but also redistribution of the conformational and oligomerization ensemble toward a stabilized dimeric assembly. We envision that our integrative mass spectrometry platform provides future development of concentration‐resolved native MS at various substoichiometric inhibitor concentrations to investigate key allosteric intermediates and/or isoforms. Key allosteric regions provided in this study could also provide a basis for future mutational studies of the dimer interface and N‐finger, correlating the structure of main protease and its oligomeric states with in vitro enzyme activity and potential therapeutic implications.

Beyond M^pro^, this work highlights the broader importance of considering oligomerization dynamics and conformational heterogeneity in the design of antiviral therapeutics targeting multimeric enzymes. The structural features identified here provide a foundation for future development of inhibitors that modulate quaternary structure and subunit exchange as complementary mechanisms of enzymatic regulation.

## AUTHOR CONTRIBUTIONS

**Syuan‐Ting Kuo:** Conceptualization; methodology; data curation; investigation; validation; writing – original draft. **Zhenyu Xi:** Methodology; investigation; writing – review and editing. **Yan J. Sheng:** Methodology; writing – review and editing. **Shiqing Xu:** Supervision; writing – review and editing; funding acquisition; methodology. **Wenshe Ray Liu:** Funding acquisition; methodology. **David H. Russell:** Methodology; supervision; funding acquisition; writing – review and editing; conceptualization. **Xin Yan:** Methodology; conceptualization; supervision; funding acquisition; writing – review and editing; writing – original draft.

## Supporting information


**FIGURE S1:** Mass shifts observed upon inhibitor binding to M^PRO^.
**FIGURE S2:** Sequence coverage map of the main protease.
**FIGURE S3:** Scheme of exchange kinetics.
**FIGURE S4:** EX1 kinetic fitting and statistics.
**FIGURE S5:** Principal component analysis of residue motions in mpro for N‐finger dynamic investigation.
**FIGURE S6:** Time‐evolved subunit exchange profile for halicin‐inhibited M^PRO^.
**FIGURE S7:** Exchange profiles of overlapping peptides spanning the N‐finger of APO M^PRO^.
**TABLE S1:** HDX‐MS data summary.

## Data Availability

The data that supports the findings of this study are available in the supplementary material of this article.
