# Supplementary material for: Structural insights into subunit exchange inhibition and ligand‐induced dimerization of SARS‐CoV‐2 main protease
Source: Protein Sci. 2026 Nov 11;35(12):e70874. doi: 10.1002/pro.70874 (PMC13752713; doi:10.1002/pro.70874)
Supplement: Supplementary file 1 [file PRO-35-e70874-s001.pdf]

Structural Mass Spectrometry Reveals Mechanisms of Subunit Exchange Inhibition and Ligand-Induced Dimerization in SARS-CoV-2 Main Protease (M^{pro})

Syuan-Ting Kuo,[a] Zhenyu Xi,[a] Yan J Sheng,[a] Shiqing Xu,[a] Wenshe Ray Liu,[a] David H. Russell*[a] and Xin Yan*[a]

Department of Chemistry, Texas A&M University, College Station, Texas 77843, USA

SUPPORTING INFORMATION

S1. PRINCIPAL COMPONENT ANALYSIS (PCA) AND K-MEANS CLUSTERING

FIGURE S1. MASS SHIFTS OBSERVED UPON INHIBITOR BINDING TO M^{PRO}

FIGURE S2. SEQUENCE COVERAGE MAP OF THE MAIN PROTEASE

FIGURE S3. SCHEME OF EXCHANGE KINETICS

FIGURE S4. EX1 KINETIC FITTING AND STATISTICS

FIGURE S5. PRINCIPAL COMPONENT ANALYSIS OF RESIDUE MOTIONS IN MPRO FOR N-FINGER DYNAMIC INVESTIGATION

FIGURE S6. TIME-EVOLVED SUBUNIT EXCHANGE PROFILE FOR HALICIN-INHIBITED M^{PRO}

FIGURE S7. EXCHANGE PROFILES OF OVERLAPPING PEPTIDES SPANNING THE N-FINGER OF APO-M^{PRO}

TABLE S1. HDX-MS DATA SUMMARY

S1. Principal component analysis (PCA) and K-means clustering. For dimensionality reduction, 1001 frames were extracted evenly from the simulation trajectory, with preliminary removal of rotational and translational motion to focus on internal conformational changes. The structures were aligned to the starting crystal structure (7JPY), and the alpha carbon coordinates of residues 1 to 17 (the N-finger region) were normalized and used as input features for PCA. The first principal component (PC1) values were subsequently used as input for K-Means clustering. Residue contributions to protein motion were quantified by calculating the normalized variance of the coordinates for each residue. The transition direction between clusters was determined by averaging the coordinates of all structures within each cluster. Other than residues Arg4, Lys5, and Met6 mentioned in main text, the analysis of PC2 and PC3 revealed minor translational contribution of Lys12 and Pro9, respectively.

(a)

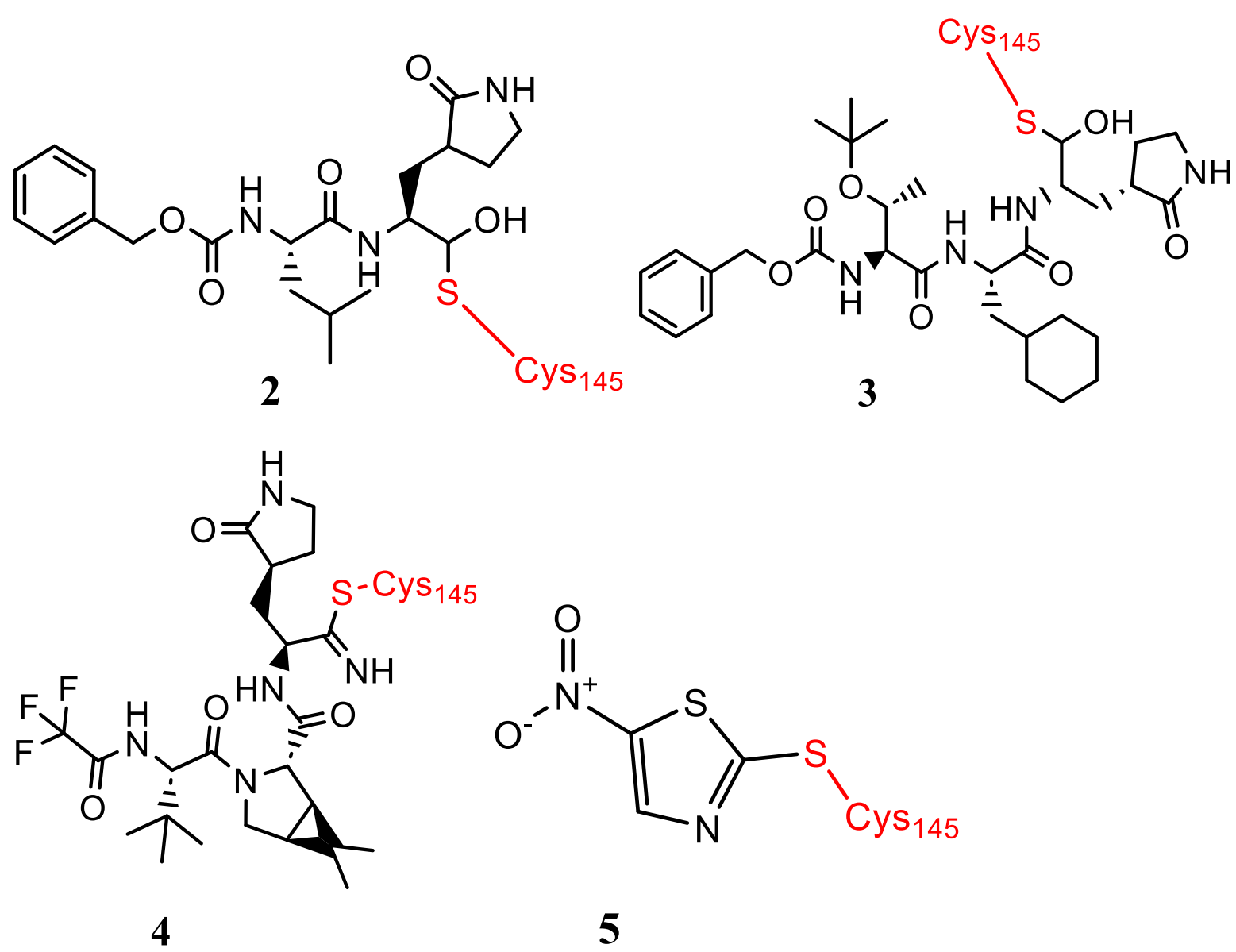

(b)

	State	Deconvoluted Mass (Da)	Unidec DScore	Experimental Ligand Mass (Da)*	Theoretical Ligand Mass (Da)	Mass Deviation (Da)
1	Apo-M^{pro}	67,599	89.6	-	-	-
2	+GC376	68,406	82.3	403.5	403.2	+0.3
3	+MPI8	68,799	87.9	600.0	600.4	-0.4
4	+Nirmatrelvir	68,595	90.9	498.0	499.2	-1.2
5	+Halicin	67,854	90.7	127.5	128.0	-0.5

* The experimental ligand mass shifts were calculated by considering two active sites were both complexed with inhibitors.

Figure S1. Mass shifts observed upon inhibitor binding to M^{pro}. (a) structures of 2-5; (b) Deconvoluted masses were determined using an open-source mass deconvolution software UniDec[4] with the following parameter settings: charge range 1-50; mass range 5,000-100,000; sample every mass (Da) 1.0; peak detection range (Da): 1; and peak detection threshold: 0.05. Theoretical ligand masses were calculated based on inhibitor binding modes 2-5 at Cys145 of M^{pro}.

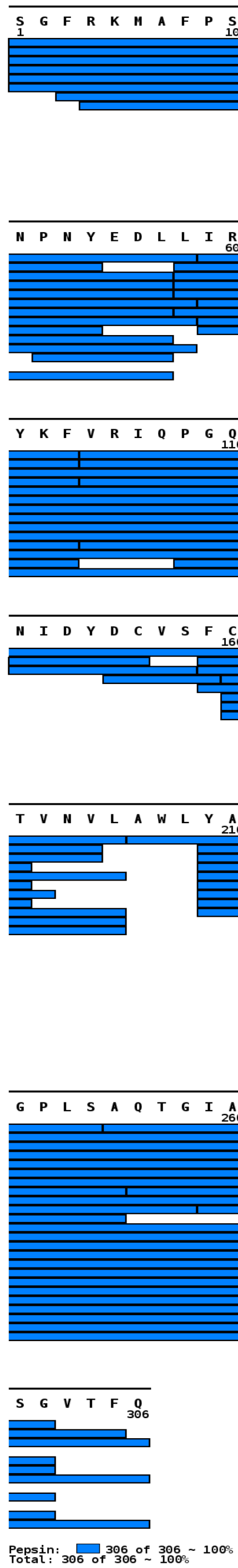

Figure S2. Sequence coverage map of the main protease. Immobilized pepsin on beads was used for digestion, generating overlapping peptides that provided complete sequence coverage. The visualization of sequence coverage map was generated by the DrawMap module in MSTools[5].

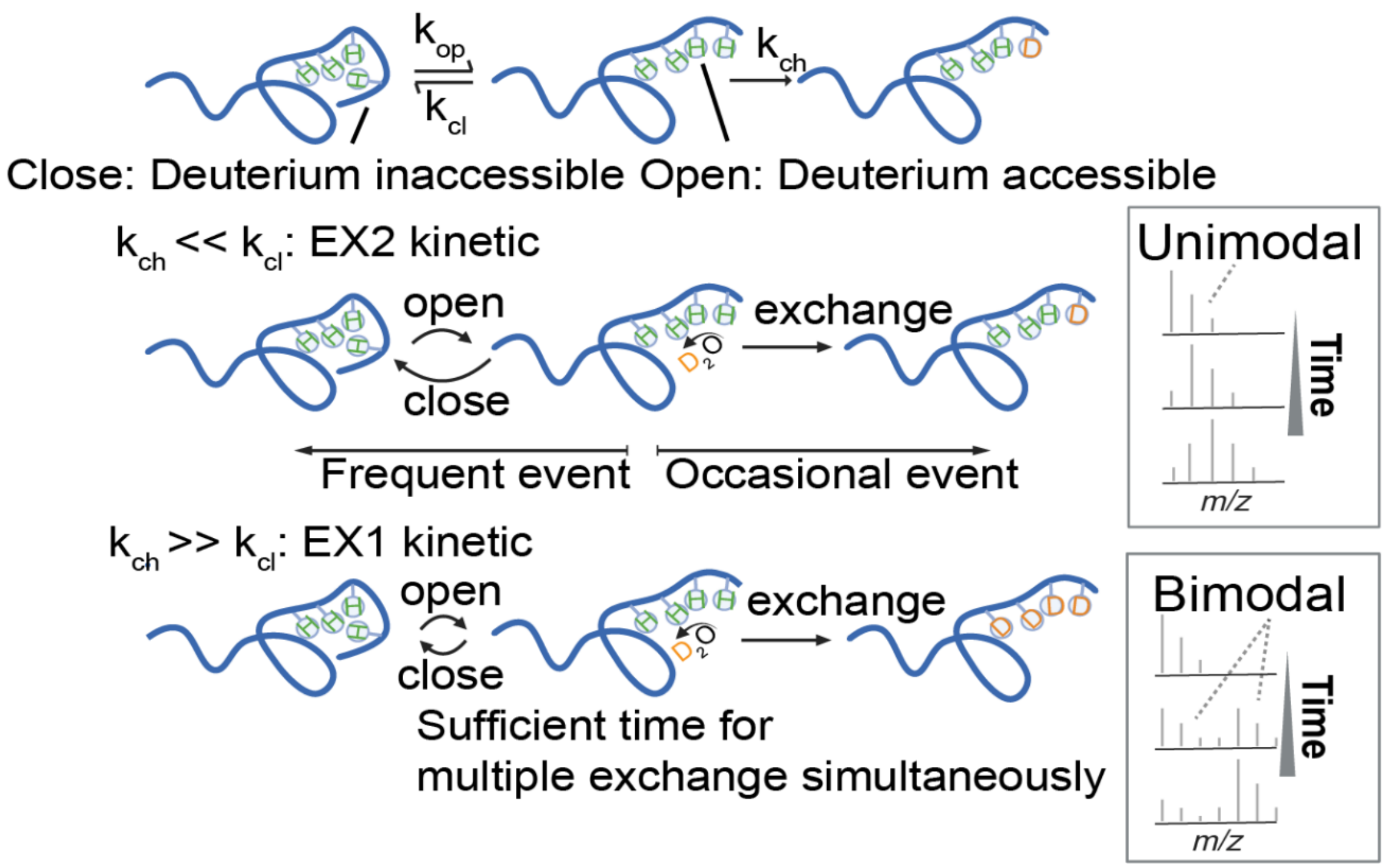

Figure S3. Scheme of exchange kinetics. The rates of opening (k_{op}), closing (k_{cl}), and intrinsic chemical exchange (k_{ch}) are shown. In the EX2 kinetic regime, backbone amide hydrogens are largely inaccessible to exchange in the 'closing' state, resulting in infrequent exchange events. In contrast, in the EX1 kinetic regime, a drastic conformational change allows the amide hydrogens to populate the 'opening' state for a sufficiently long duration, enabling simultaneous exchange events.

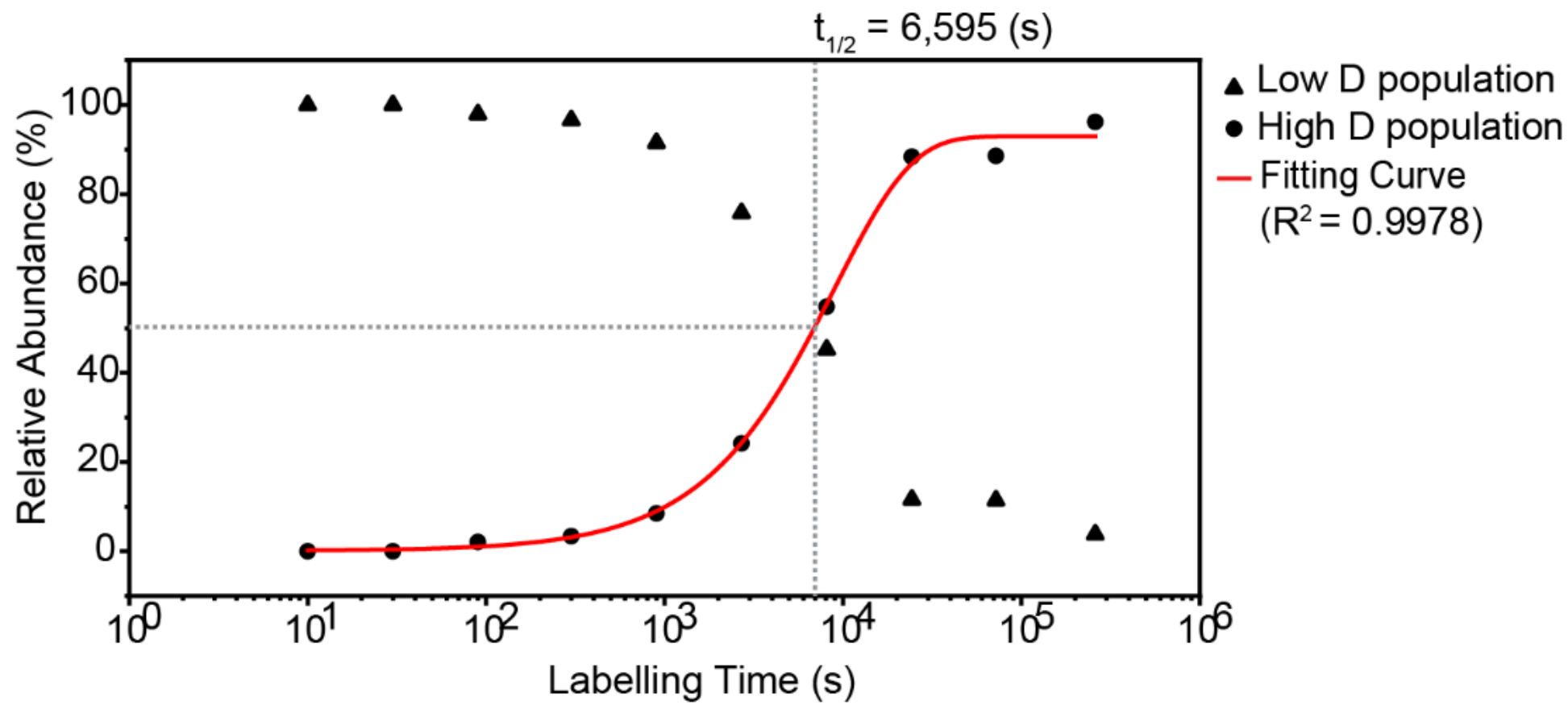

Figure S4. EX1 kinetic fitting and statistics. The high-deuterium (high-D) uptake population (%) was modelled using a single exponential-growth equation as:

$$A_t = A_0 e^{\frac{t}{t_1}} + C_0$$

Here, A_t represents the relative abundance of high-D population and t denotes the labelling time. The fitting results and parameters presented in the table are used to calculate the half-life ($t_{1/2}$) of conversion discussed in the main text.

	C_0	A_0	t_1	Reduced Chi-Sqr	R-Square (COD)
Value	92.94681	92.84276	8943.8566	4.82516	0.99778
Standard Deviation	1.429	1.68995	571.01908	-	-

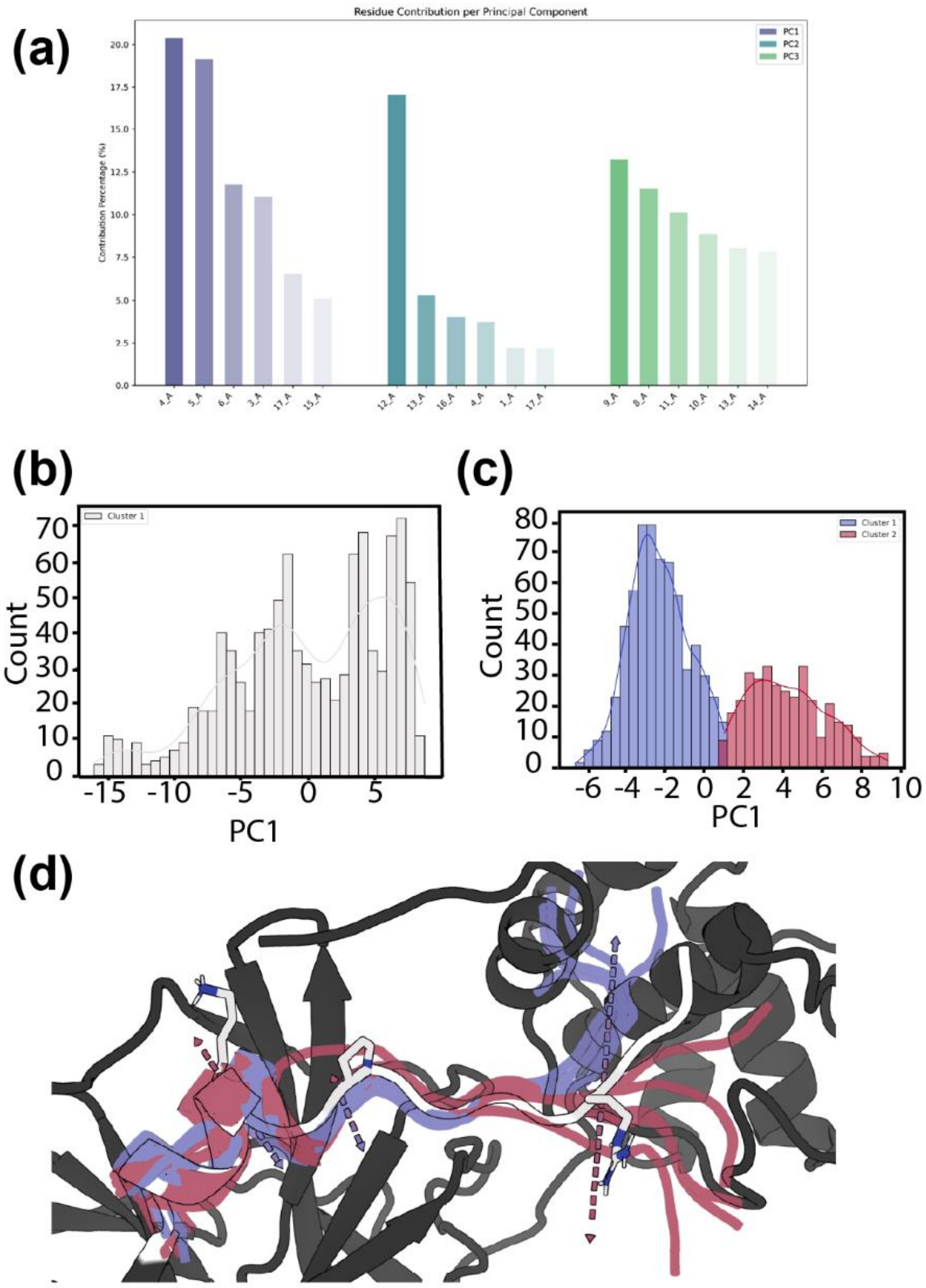

Figure S5. Principal component analysis of residue motions in M^{pro} for N-finger dynamic investigation. PCA was performed on the Cartesian coordinates of residues 1–17 across the simulation trajectory. Briefly, a covariance matrix was constructed from the fluctuations of each residue's coordinates relative to their average positions, and this matrix was diagonalized to yield a set of orthogonal principal components (eigenvectors) ranked by the fraction of total variance they explain. Each residue's contribution to a given principal component is reflected in the magnitude of its corresponding eigenvector coefficient (loading). Because the dominant principal components (e.g., PC1, PC2) capture the largest-amplitude collective motions in the system, residues with the highest loadings on these components can, where applicable, be physically interpreted as those undergoing the most significant positional changes or movement. **(a)** Projection of molecular dynamics trajectories onto the first three principal components. **(b)** Contribution of individual residues to PC1 in dimeric **M^{pro}**. **(c)** Contribution of individual residues to PC1 in monomeric **M^{pro}**. Notably, two distinct clusters, identified by K-means classification, were observed in monomeric **M^{pro}** (c) but not in dimeric **M^{pro}** (b). **(d)** Representative N-finger conformers color-coded according to K-means cluster assignment. Arrows indicate the direction and magnitude of residue displacements relative to their initial positions.

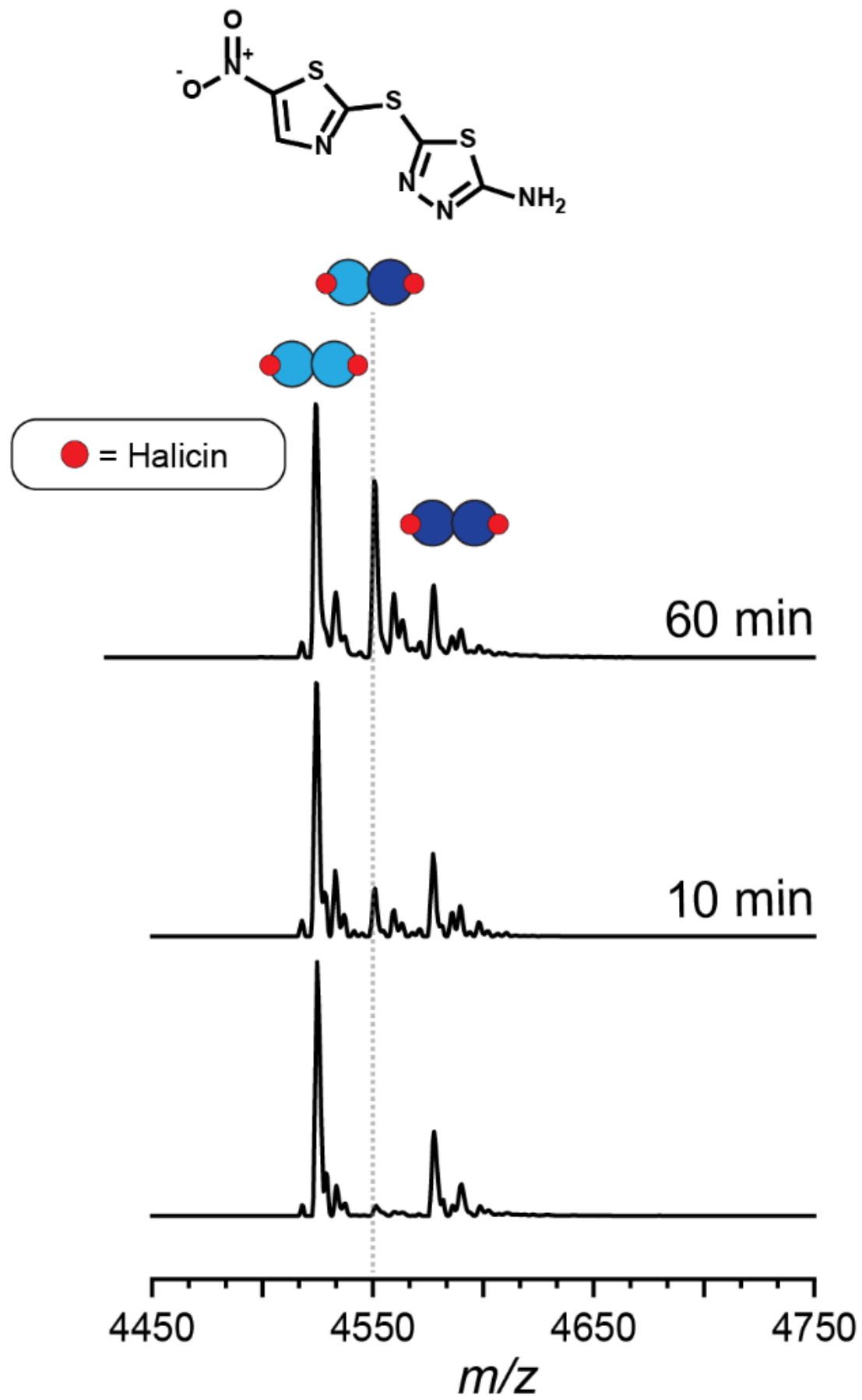

Figure S6. Time-evolved subunit exchange profile for halicin-inhibited M^{pro}. Halicin-treated dimers retained measurable subunit exchange within 60 minutes. Accurate quantification in the presence of halicin proved challenging, as saturation of both active sites required a higher compound-to-protein ratio (20 μM halicin per 5 μM protein). At this elevated concentration, halicin substantially suppressed ionization efficiency of M^{pro}, resulting in signal intensities that deviated from the expected 1:1 ratio even when equimolar protein concentrations were used.

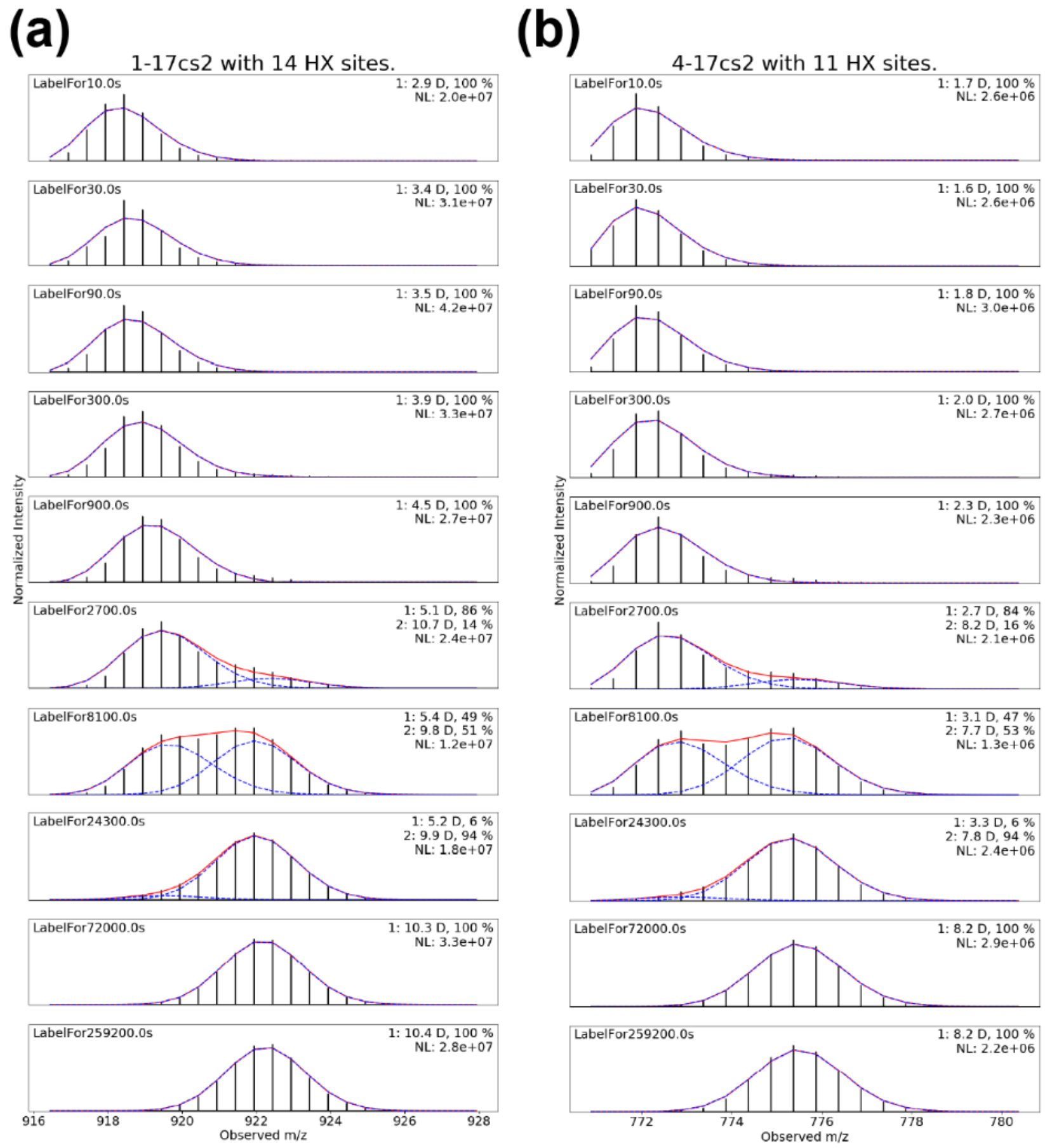

Figure S7. Exchange profiles of overlapping peptides spanning the N-finger of apo-M^{pro}, corresponding to residues (a) 1–17 and (b) 4–17 for confirmation of EX1 kinetics.

Table S1. HDX-MS data summary.

**Sequence coverage**	100% (306/306 residues)
**Number of peptides**	260
**Redundancy (avg. peptides/residue)**	13.8
**Average peptide length**	16.2 residues (range 5-70)
**Number of replicates**	1 biological replicate; 10 time points
**Back-exchange estimate**	~26% (mean 25.9 +/- 8.9% SD; median 25.3%), calculated from all-D control (n=253 peptides)

REFERENCES

(1) Yang, K. S.; Alex Kuo, S.-T.; Blankenship, L. R.; Geng, Z. Z.; Li, S. G.; Russell, D. H.; Yan, X.; Xu, S.; Liu, W. R. *Current Research in Chemical Biology* **2022**, *2*, 100025. DOI: https://doi.org/10.1016/j.crchbi.2022.100025.

(2) Qiao, Y.; Yu, G.; Kratch, K. C.; Wang, X. A.; Wang, W. W.; Leeuwon, S. Z.; Xu, S.; Morse, J. S.; Liu, W. R. *Journal of the American Chemical Society* **2020**, *142* (15), 7047-7054. DOI: 10.1021/jacs.0c00252.

(3) Kan, Z.-y.; Ye, X.; Skinner, J. J.; Mayne, L.; Englander, S. W. *Analytical Chemistry* **2019**, *91* (11), 7474-7481. DOI: 10.1021/acs.analchem.9b01682.

(4) Marty, M. T.; Baldwin, A. J.; Marklund, E. G.; Hochberg, G. K.; Benesch, J. L.; Robinson, C. V. *Anal Chem* **2015**, *87* (8), 4370-4376. DOI: 10.1021/acs.analchem.5b00140 From NLM Medline.

(5) Kavan, D.; Man, P. *International Journal of Mass Spectrometry* **2011**, *302* (1-3), 53-58. DOI: 10.1016/j.ijms.2010.07.030.

(6) Zhang, Z.; Li, W.; Logan, T. M.; Li, M.; Marshall, A. G. *Protein Sci* **1997**, *6* (10), 2203-2217. DOI: 10.1002/pro.5560061015 From NLM Medline.
